# The influence of minimum dietary diversity on undernutrition among children aged 6–23 months in Ethiopia: a multilevel mixed-effect analysis based on 2019 Ethiopian mini demographic and health survey

**DOI:** 10.3389/fpubh.2024.1436683

**Published:** 2024-10-09

**Authors:** Abdu Hailu Shibeshi, Zeytu Gashaw Asfaw

**Affiliations:** ^1^Department of Statistics, College of Natural and Computational Science, Samara University, Samara, Ethiopia; ^2^Department of Epidemiology and Biostatistics, School of Public Health, Addis Ababa University, Addis Ababa, Ethiopia

**Keywords:** dietary diversity, undernutrition, multilevel logistic regression, children, Ethiopia

## Abstract

**Background:**

Undernutrition persists as a critical issue in developing countries like Ethiopia due to poor feeding practices for infants and young children. The impact of dietary diversity on children’s health in Ethiopia remains unclear, necessitating further investigation to develop effective prevention strategies.

**Objective:**

To examine the association between minimum dietary diversity and undernutrition among children aged 6–23 months in Ethiopia.

**Methods:**

Data from the 2019 Ethiopian Mini Demographic and Health Survey, including 1,501 women with children aged 6–23 months, were analyzed using STATA version 17 software. Child stunting, wasting, and underweight were assessed using Z-scores. Dietary diversity was measured using minimum dietary diversity. Multilevel logistic regression analysis determined associations, presenting results as crude odds ratios (COR) and adjusted odds ratios (AOR) with 95% confidence intervals (CI).

**Results:**

Overall, 10.99% of children had adequate minimum dietary diversity, with Addis Ababa (44.57%) and Somalia (1.47%) showing the highest and lowest prevalences, respectively. The highest stunting prevalence was in the Amhara region (45.86%), while Addis Ababa had the lowest (9.78%). Wasting was lowest in Addis Ababa (1.09%) and highest in Tigray (17.07%). Underweight prevalence ranged from 2.17% in Addis Ababa to 33.33% in Tigray. Children with adequate minimum dietary diversity (MDD) had significantly lower odds of stunting (AOR = 0.68, 95% CI = 0.45, 0.96), underweight (AOR = 0.51, 95% CI = 0.27, 0.99), and wasting (AOR = 0.40, 95% CI = 0.17, 0.97) compared to those who had inadequate minimum dietary diversity (MDD).

**Conclusion:**

This study highlights the association between minimum dietary diversity and stunting, wasting, and underweight among Ethiopian children aged 6–23 months. Urgent nutrition-specific interventions are needed, particularly in regions with high undernutrition rates and low dietary diversity. Targeted interventions focusing on promoting diverse and nutritious diets for children, along with improving access to essential healthcare services, are imperative to mitigate the burden of undernutrition and ensure the well-being of Ethiopia’s youngest population and reinforcing existing programs is crucial to address this public health issue effectively.

## Background

Nutritional status, beyond mere calorie intake, encompasses an individual’s access to and absorption of essential nutrients, reflecting their health and well-being (1, 2). Dietary diversity can be considered as an outcome of the nutritional status of children (3–19) and a key component of high-quality diets (20–22). More than just a measurement, dietary diversity plays a crucial role in shaping the health of young children. It reflects the quality of their diet, ensures they get the essential micronutrients they need, and indicates their access to a variety of nutritious foods (23–26). Ultimately, the diversity of their diet directly impacts their health outcomes (16, 27).

Undernutrition significantly hinders public health in developing countries like Ethiopia. Dietary diversity, consuming a variety of food groups, is recognized as a key indicator of dietary quality and micronutrient adequacy. Studies have found a clear link between inadequate dietary diversity and the prevalence of undernutrition, including stunting, underweight, and wasting, which hinder children’s physical and cognitive development (12, 16, 28). Despite the World Health Organization’s (WHO) recommendation for young children to consume a minimum of four diverse food groups for optimal development (grains, roots, legumes, dairy, flesh foods, eggs, vitamin A-rich fruits, and other fruits/vegetables), a concerning reality persists in developing countries. Studies reveal widespread inadequate dietary diversity among children in these regions (6–8, 29), with a UNICEF report highlighting that less than 25% of children aged 6–23 months in LMICs meet the minimum dietary diversity and meal frequency standards (4, 5). In lower-middle income counties (LMICs), limited access to nutritious food creates a critical dietary diversity gap. This not only hinders children’s physical growth but also harms their cognitive development and future health, highlighting the urgent need for multifaceted solutions.

Despite being a cornerstone of Sustainable Development Goal 2: Zero Hunger by 2030, achieving diverse and nutritious diets for children remains a daunting challenge in low and middle-income countries (LMICs). Poverty, limited resource access, and inadequate infrastructure leave many parents struggling to offer the variety of foods essential for their children’s optimal growth and development (29–38). While the consequences of limited dietary diversity are profound, it’s important to recognize its complex relationship with child nutrition. Studies reveal that low dietary diversity not only reflects existing nutritional deficiencies but also fuels further vulnerabilities (36). Children with restricted dietary intake are significantly more prone to nutrient deficiencies, impacting their physical and cognitive development (32, 34, 35, 38). Conversely, a diverse diet rich in essential nutrients forms the bedrock of good health, as evidenced by (36) findings on healthy growth patterns in children with varied diets.

Undernutrition remains a critical public health concern in Ethiopia, plaguing nearly 37% of children under five, particularly in the form of stunting (39). This alarming prevalence underscores the need for a comprehensive understanding of the factors influencing child nutrition. Minimum Dietary Diversity (MDD), defined as the consumption of foods from four or more food groups within a given period, serves as a key indicator of Infant and Young Child Feeding (IYCF) practices and plays a crucial role in preventing undernutrition (40).

Ethiopia grapples with high rates of undernutrition, with 37% of children under five stunted and 10% wasted (10). Limited dietary diversity is prevalent, with only 14% of children aged 6–23 months meeting the minimum dietary diversity standards (9). Previous research in Ethiopia highlights associations between low dietary diversity and undernutrition in children (8, 13). Despite numerous studies on the link between minimum dietary diversity (MDD) and undernutrition in Ethiopian children (29, 33, 36, 37), crucial knowledge gaps remain. Existing research primarily employs national or regional analyses, neglecting the intricate interplay between individual, household, and community factors influencing MDD and undernutrition (33). Additionally, the focus falls mainly on individual-level determinants like maternal education, limiting insights into how community-level variables (residence, geographical region) and household-level factors (wealth index, toilet facility, and drinking water source) shape MDD and various forms of undernutrition (stunting, wasting, underweight) (36). Furthermore, intervention-specific insights, crucial for tailoring strategies to address disparities, are largely absent (29).

The age range of 6–23 months is considered critical for assessing minimum dietary diversity because during this period, infants and young children transition from exclusive breastfeeding to consuming a variety of solid foods. This window is crucial for ensuring adequate nutrition and preventing under-nutrition, as it’s a period of rapid growth and development where nutrient requirements are high. Therefore, understanding dietary diversity within this age group provides insights into nutritional practices and potential interventions to address under-nutrition among young children.

Therefore, by conducting a multilevel mixed-effect logistic regression analysis using the rich data of the 2019 Ethiopian mini-EDHS, this research will address these gaps by: (1) examining the association between MDD and different types of undernutrition in children aged 6–23 months and (2) assessing the associated factors between individual, household, and community level and undernutrition. This comprehensive approach, addressing critical research gaps highlighted in existing literature, will significantly contribute to improving child nutrition in Ethiopia through evidence-based interventions and public health strategies.

## Methods

### Data source, study setting, design, and population

This study included a secondary data analysis based on data from the Mini Demographic Health Surveys (DHSs). Mini Demographic and Health Survey (EMDHS) 2019 is the second Mini Demographic and Health Survey conducted in Ethiopia. Data collected through the 2019 EMDHS is intended to help decisionmakers and program managers evaluate and plan programs and strategies to improve the health of the nation’s population. The sample used in the survey is a series of population censuses (EAs) conducted by the Ethiopian Population and Housing Census (EPHC) and conducted by the Statistics Agency (CSA) (15).

The survey used a cross-sectional design relying on a two-stage stratified sampling technique to recruit participants. In the first stage, 305 EA (93 urban and 212 rural) were selected with the proportional probability of EA size (based on EPHC 2019 classification). In the second round of sampling, a random number of 30 households per group were selected with equal chance of selection from the new list of households (15).

**Figure 1:**
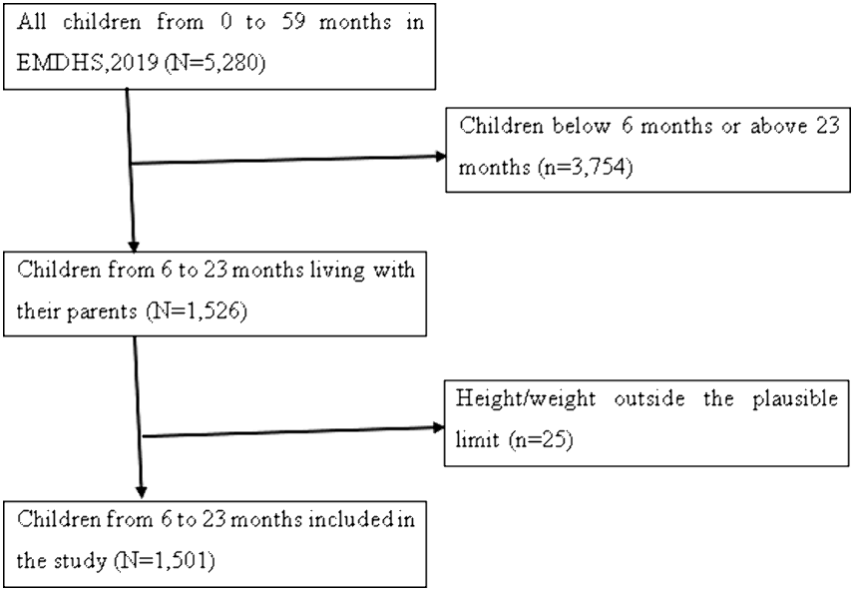
Selection of children aged 6–23 months from EMDHS 2019, 2024.

The surveys cover a variety of demographic, household, and key health indicators. For this study, we focused on the Kids Record (KR) file, which contains detailed information about children under the age of five. After applying the criteria, the final analysis was based on a weighted sample of 1,501 children ages 6–23 months (Table 1). Detailed information about the DHS methodology can be found in the official database https://dhsprogram.com/Methodology/index.cfm. We excluded missing values (children outside the 6–23 age range) and values that were not possible in our study as shown in (Figure 1).

**Table 1:** Description of the study samples for each region.

Region	Weighted sample (*n*)	Percentage (%)
Tigray	123	8.19
Afar	160	10.66
Amhara	157	10.46
Oromia	178	11.86
Somali	136	9.06
Benishangul Gumuz	135	8.99
South Nation Nationality and Peoples of Republic	177	11.79
Gambela	116	7.73
Harari	111	7.40
Addis Ababa	92	6.13
Dire Dawa	116	7.73

### Study variables and measurements

#### Outcome variable

The study used three indicators to measure the outcome variable (undernutrition): stunting, wasting, and underweight. Child growth failure was used to assess these indicators, which were classified based on height-for-age z-score (HAZ), weight-for-age z-score (WAZ), and weight-for-height z-score (WHZ) as defined by the WHO Growth Reference Standard (17). According to the WHO Growth reference standard, any child aged 6–23 months whose HAZ, WAZ, and WHZ fell below minus 2 (−2.0) standard deviations (SDs) lower than the mean on the reference standard for a given age was classified as stunted, underweight, and wasted, respectively. Those who were stunted, underweight, and wasted were coded as “1,” while those who were normal were coded as “0.”

#### Explanatory variables

Minimum Dietary Diversity (MDD) is a measure of dietary diversity and was the most important factor identified in this study. MDD is defined as the proportion of children aged 6–23 months who consume four or more of the seven food groups (12, 14, 16, 36, 41, 42). The seven food groups include grains, roots, and legumes; legumes and nuts; dairy products (milk, yoghurt, cheese); meat products (meat, fish, chicken, liver or other organs); Eggs; Fruits and vegetables rich in vitamin A; and other fruits and vegetables. To predict MDD, all food groups were summed with a score ranging from 0 to 7. Each child who ate any of the food groups was given a score from (1) to zero (0). eating a food group Children who ate at least four (≥4) of the food groups were reported to have adequate MDD and were coded as “1,” and children who consumed less than four food groups were coded as “0 = inadequate.” The classification and categorization mentioned previously were based on literature that studied either dietary diversity alone or its association with undernutrition (14, 41–44).

In addition to MDD, researchers considered other factors that might influence childhood undernutrition. These factors, chosen based on previous studies (6, 10, 13, 31, 32, 34, 35, 38) and available data from the Mini Demographic and Health Survey (MDHS), were grouped into three categories: individual-level, household-level, and community-level variables. The **individual-level variables:** This category included characteristics of the child and the mother, including sex of the child, age of child, birth order, mother’s age, maternal educational level, number of antenatal care attendants (ANC), place of delivery, and marital status. **Household-level variables:** This category included factors like drinking water source, toilet facility, and wealth index. The community-level variables: This category included place of residence and geographical regions.

#### Data management and statistical analysis

To ensure their analysis accurately represented the real population, the researchers considered factors like sampling weight, the primary sampling unit (PSU), and strata. Sampling weights allowed them to calculate precise standard errors.

For data management, generating summary statistics (descriptive statistics), and performing the analysis, we used the STATA 17 statistical software. Since the data came from the Demographic and Health Survey (DHS), which has a hierarchical structure (individuals nested within households, and households within communities), we employed a multilevel binary logistic regression model. This approach lets them analyze how individual characteristics, household factors, and community-level variables are all associated with the likelihood of undernutrition. Bivariable multilevel binary logistic regression analysis was conducted to identify the variables eligible for multivariable analysis. Variables with a *p*-value less than 0.20 in this initial analysis and those found important in the literature were considered candidates for multivariable multilevel binary logistic regression analysis.

### Model building and parameter estimation method

Indicators of undernutrition (stunting, wasting, and underweight) are a binary outcome $Y_{ij}$ from the sample of individuals j=1, from a set of cluster/EA i , a set of individual exposures $\left\{Z_{iju},\;u=1,2\right\}$ and a set of cluster-level exposures $\left\{X_{ir},\;r=1,2\right\}$ .

$$Y_{ij}∼\mathrm{Bernoulli}\;\left(P_{ij}\right)$$

Null model: $\mathrm{logit}\left(P_{ij}\right)=\mu +u_{i}$

Model 1: $\mathrm{logit}\left(P_{ij}\right)=\mu +\beta X_{ij}+u_{i}$

Mode1 2: $\mathrm{logit}\left(P_{ij}\right)=\mu +\gamma Z_{i}+u_{i}$

Model 3: $\mathrm{logit}\left(P_{ij}\right)=\mu +\beta X_{ij}+\gamma Z_{i}+u_{i}$

We explored the variability across communities (clusters) in undernutrition prevalence among children aged 6–23 months using three measures: the Likelihood Ratio (LR) test, Intra-class Correlation Coefficient (ICC), and Median Odds Ratio (MOR). The ICC specifically quantifies the proportion of individual variation in undernutrition that can be attributed to differences between communities (18).

$ICC=\sigma^{2}/\left(\sigma^{2}+\pi^{2}/3\right)$

The Median Odds Ratio (MOR) is a statistical measure that quantifies the variation or heterogeneity in children’s undernutrition between clusters in terms of the odds ratio scale. It is defined as the median value of the odds ratio between the cluster with a high likelihood of children undernutrition and the cluster at lower risk when randomly picking out individuals from two clusters (EAs) (45).


$$\mathrm{MOR}=\exp\;\sqrt{\;\left(2*\partial^{2}*0.6745\right)}\;\sim \mathrm{MOR}=\exp\;\left(0.95*\partial \right)$$

∂ ^2^ indicates the cluster variance.

Bivariable multilevel binary logistic regression analysis was conducted to identify variables eligible for multivariable analysis ( p≤0.2 ). Four models were constructed for multivariable multilevel binary logistic regression. The first was a null model without explanatory variables to determine the extent of cluster variation in children’s undernutrition. The second model was fitted with individual-level variables, the third with community-level variables, and the fourth with both individual and community-level variables simultaneously. Deviance was used to verify the model’s fitness, and the model with the lowest deviance was considered the best-fit model. Finally, the adjusted odds ratio (AOR) with 95% confidence interval (CI) were reported, and variables with a p−value≤0.05 in the multivariable analysis were considered statistically significant predictors.

## Results

### Descriptive characteristics of the study participants

The distribution of undernutrition among the 1,501 children who were included in this study was presented in Table 2. According to the findings, 31.08% of children who had inadequate dietary diversity experienced stunting. Furthermore, 22.12% of the children who had inadequate dietary diversity were underweight, whereas 10.25% were wasted. In terms of child traits, male children were found to be more stunted (32.38%), wasted (10.05%), and underweight (23.11%) compared to female children. Based on the maternal characteristics data, children with mothers in the 30–34 age range had the highest prevalence of stunting (35.69%), followed by children with mothers in the same age range who had the highest prevalence of underweight (25.25%).

**Table 2:** Socio-demographic characteristics and distribution of under-nutrition among children aged 6–23 months in Ethiopia, 2024 (*n* = 1,501).

Variables	Weighted *n* (%)	Stunted %	Wasted %	Underweight %
Minimum dietary diversity				
Inadequate	1,171 (78.01)	364 (31.08)	120 (10.25)	259 (22.12)
Adequate	330 (21.99)	79 (23.94)	14 (4.24)	32 (9.70)
Child characteristics				
Sex of child				
Male	766 (51.03)	248 (32.38)	77 (10.05)	177 (23.11)
Female	735 (48.97)	195 (26.53)	57 (7.76)	114 (15.51)
Age of child (in months)				
6–8	264 (17.59)	49 (18.56)	20 (7.58)	39 (14.77)
9–11	239 (15.92)	63 (26.36)	22 (9.21)	42 (17.57)
12–17	561 (37.38)	161 (28.70)	52 (9.27)	100 (17.83)
18–23	437 (29.11)	170 (38.90)	40 (9.15)	110 (25.17)
Birth order				
1–3	884 (58.89)	248 (28.05)	61 (6.90)	148 (16.74)
4–6	432 (28.78)	139 (32.18)	52 (12.04)	98 (22.69)
7 and above	185 (12.33)	56 (30.27)	21 (11.35)	45 (24.32)
Maternal characteristics				
Mother’s age				
15–19	107 (7.13)	34 (31.78)	10 (9.35)	26 (24.03)
20–24	349 (23.25)	97 (27.79)	28 (8.02)	52 (14.90)
25–29	509 (33.91)	140 (27.50)	40 (7.86)	88 (17.29)
30–34	297 (19.79)	106 (35.69)	29 (9.76)	75 (25.25)
35–39	161 (10.73)	48 (29.81)	18 (11.18)	34 (21.12)
40–44	60 (4.00)	13 (21.67)	6 (10.00)	13 (21.67)
45–49	18 (1.20)	5 (27.78)	3 (16.67)	3 (16.67)
Maternal educational level				
No education	734 (48.90)	243 (33.11)	85 (11.58)	170 (23.16)
Primary Secondary	525 (34.98) 137 (9.13)	162 (30.86) 28 (20.44)	35 (6.67) 10 (7.03)	99 (18.86) 17 (12.41)
Higher	105 (7.00)	10 (9.52)	4 (3.81)	5 (4.76)
ANC				
None	367 (24.45)	119 (32.43)	49 (13.35)	89 (24.25)
1–3	474 (31.58)	130 (27.43)	44 (9.28)	96 (20.25)
4 or more	660 (43.97)	194 (29.39)	41 (6.21)	106 (16.06)
Place of delivery				
Home	648 (43.17)	215 (33.18)	72 (11.11)	156 (24.07)
Health facility	799 (53.23)	221 (27.66)	55 (6.88)	125 (15.64)
Others	54 (3.60)	7 (12.96)	7 (12.96)	10 (18.52)
Marital status				
Single	8 (0.53)	2 (25.00)	1 (12.50)	1 (12.50)
Married	1,419 (94.54)	416 (29.32)	127 (8.95)	273 (19.24)
divorced/widowed/separated	74 (4.93)	25 (33.78)	6 (8.11)	17 (22.97)
Household characteristics				
Drinking water source				
Improved	833 (55.50)	230 (27.61)	72 (8.64)	140 (16.81)
Unimproved	668 (44.50)	213 (31.89)	62 (9.28)	151 (22.60)
Toilet facility				
Improved	768 (51.17)	208 (27.08)	65 (8.46)	128 (16.67)
Unimproved	733 (48.83)	235 (32.06)	69 (9.41)	163 (22.24)
Wealth index				
Poorest	457 (30.45)	146 (31.95)	66 (14.44)	118 (25.82)
Poorer	245 (16.32)	87 (35.51)	23 (9.39)	58 (23.67)
Middle	219 (14.59)	76 (34.70)	11 (5.02)	37 (16.89)
Richer	202 (13.46)	72 (35.64)	16 (7.92)	38 (18.81)
Richest	378 (25.18)	62 (16.40)	18 (4.76)	40 (10.58)
Contextual-level factors				
Place of residence				
Urban	394 (26.25)	72 (18.27)	27 (6.85)	60 (15.23)
Rural	1,107 (73.75)	371 (33.51)	107 (9.67)	231 (20.87)
Geographical regions				
Tigray	123 (8.19)	46 (37.40)	21 (17.07)	41 (33.33)
Afar	160 (10.66)	59 (36.88)	20 (12.50)	35 (21.88)
Amhara	157 (10.46)	72 (45.86)	13 (8.28)	47 (29.94)
Oromia	178 (11.86)	45 (25.28)	7 (3.93)	18 (10.11)
Somali	136 (9.06)	26 (19.12)	20 (14.71)	23 (16.91)
Benishangul Gumuz	135 (8.99)	47 (34.81)	11 (8.15)	33 (24.44)
SNNPR	177 (11.79)	56 (31.64)	12 (6.78)	30 (16.95)
Gambela	116 (7.73)	24 (20.69)	17 (16.66)	28 (24.14)
Harari	111 (7.40)	32 (28.83)	5 (4.50)	15 (13.51)
Addis Ababa	92 (6.13)	9 (9.78)	1 (1.09)	2 (2.17)
Dire Dawa	116 (7.73)	27 (23.28)	7 (6.03)	19 (16.38)

However, children with mothers in the 45–49 age range had the highest prevalence of wasting (16.67%). Additionally, children of mothers with no formal education were more likely to be stunted, underweight, and wasted compared to children whose mothers had some level of education. Concerning household-level characteristics, children drinking from unimproved water sources were more likely to experience underweight (22.60%), wasting (9.28%), and stunting (31.89%) compared to those with access to improved water. The study revealed that children living in rural areas faced a higher risk of stunting (33.51%), underweight (20.87%), and wasting (9.67%) compared to their urban counterparts (Table 2).

### Prevalence of minimum dietary diversity, stunting, wasting, and underweight among children aged 6–23 months in Ethiopia

**Figure 2:**
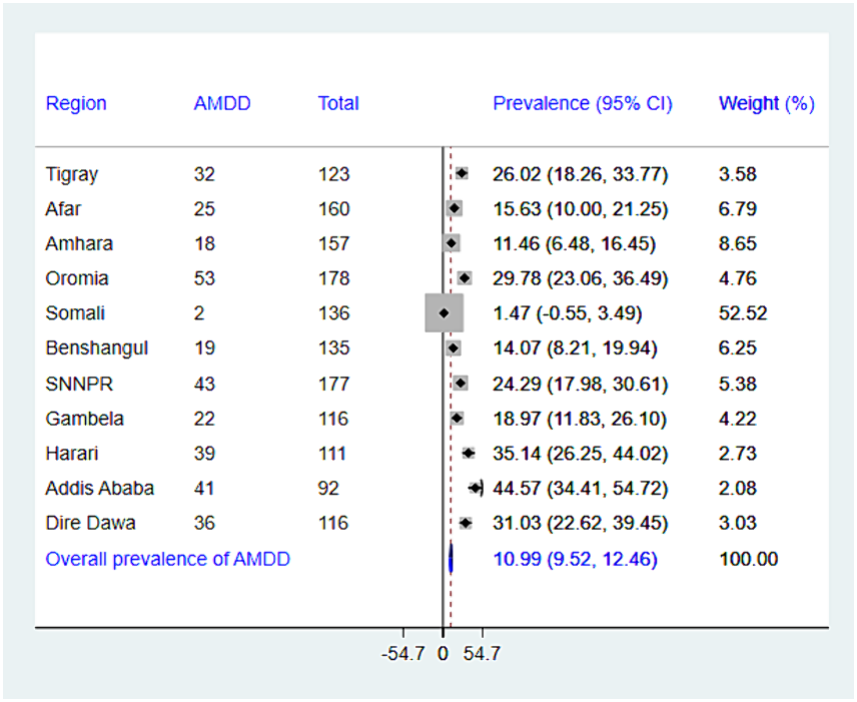
The forest plot presenting the prevalence of adequate minimum dietary diversity among children aged 6–23 months in Ethiopia, 2024.

**Figure 3:**
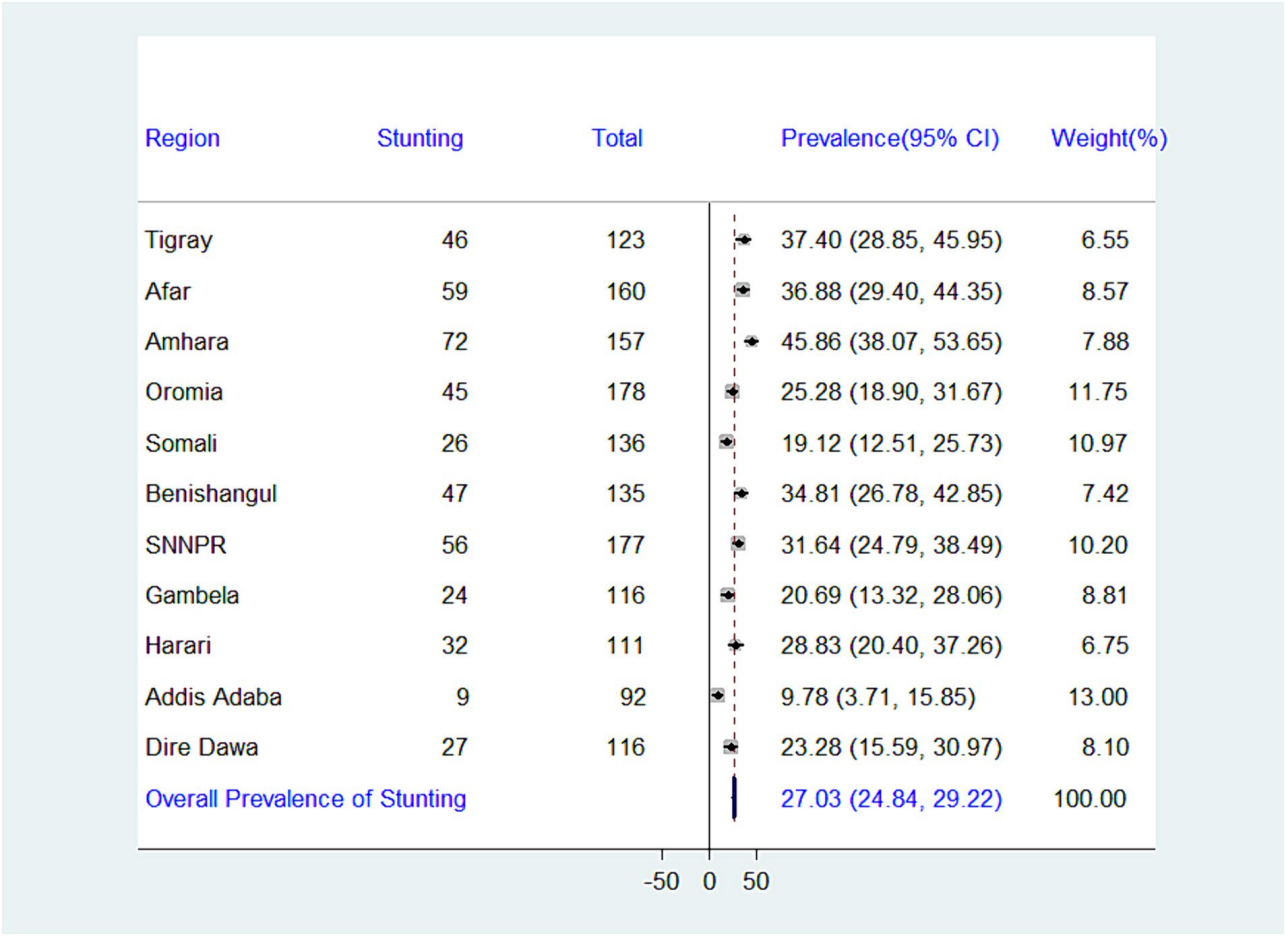
Forest plot presenting the prevalence of stunting among children aged 6–23 months in Ethiopia, 2024.

**Figure 4:**
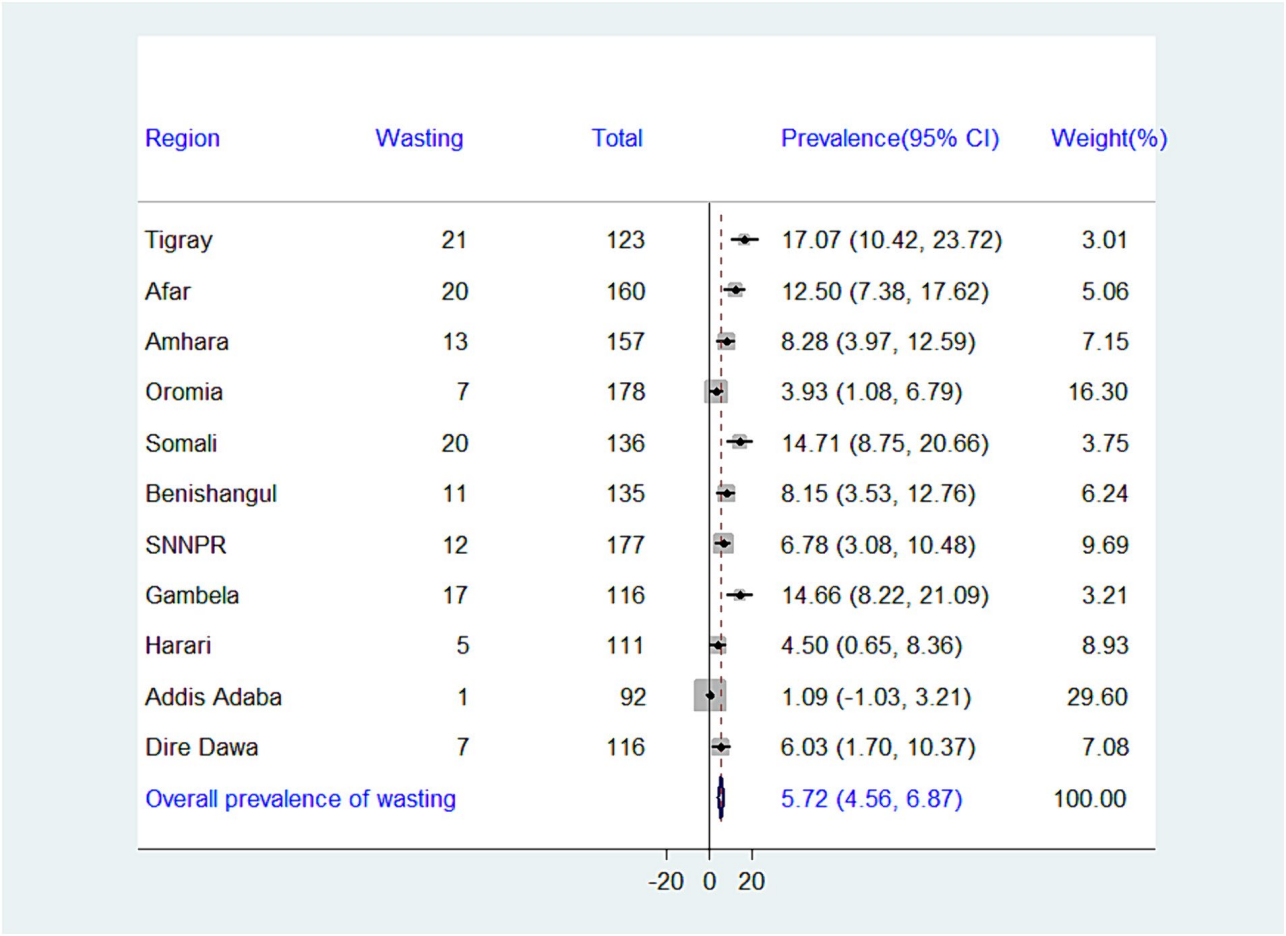
Forest plot presenting the prevalence of wasting among children aged 6–23 months in Ethiopia, 2024.

**Figure 5:**
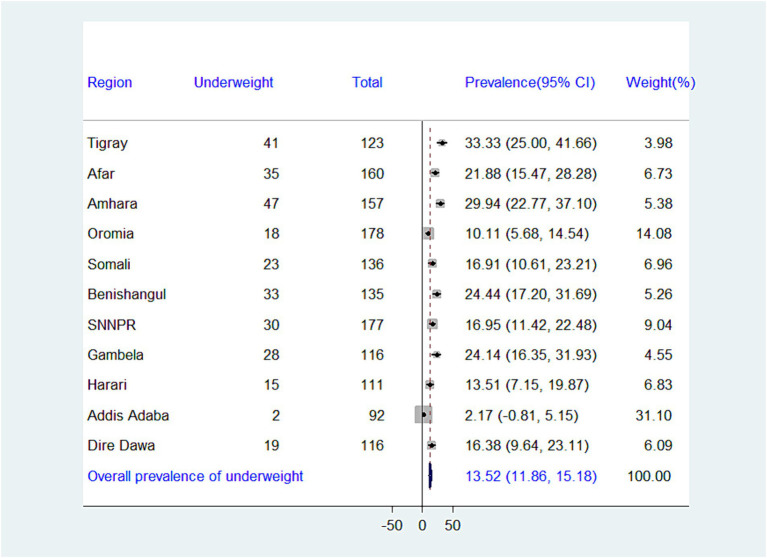
Forest plot presenting the prevalence of underweight among children aged 6–23 months in Ethiopia, 2024.

While the national prevalence of adequate minimum dietary diversity (MDD) is low (10.99%), there’s a significant difference between regions. Addis Ababa has the highest MDD (44.57%), while Somalia has the lowest (1.47%) (Figure 2). There is significant variation in the prevalence of undernutrition across regions in Ethiopia. The highest prevalence of stunting was recorded in the Amhara region (45.86%), whereas the lowest prevalence was found in Addis Ababa (9.78%), with an overall regional prevalence of 27.03% (Figure 3). The prevalence of wasting in all regions was found to be 5.72%. Addis Ababa recorded the lowest prevalence of wasting (1.09%), whereas Tigray had the highest prevalence (17.07%) (Figure 4). Lastly, the prevalence of underweight ranged from 33.33% in Tigray to 2.17% in Addis Ababa, with an overall regional prevalence of 13.52% (Figure 5).

### Model comparison and random effect analysis

Table 3 presents the comparison of models based on the lowest deviance for undernutrition, as well as the results of ICC and MOR in the null model. The results showed that the ICC in the null model for stunting was 14%, meaning that differences between clusters/EAs accounted for 14% of the overall variability for experiencing stunting. Moreover, there was variation amongst clusters, as evidenced by the null model’s MOR for stunting, which was 1.99. An individual in the cluster with a higher risk of stunting has a 1.99 times greater chance of having stunting than an individual in the cluster with a lower risk of stunting, if we were to randomly select an individual from each of the two clusters. The model with the lowest deviance value (1,630.06) was selected as the best-suited one (Model 3—including all explanatory variables) (Table 3).

**Table 3:** Model comparison and random effect results.

Stunting				
	Null model	Model 1	Model 2	Model 3
Model fitness				
LR	−896.19	−831.41	−866.63	−815.03
Deviance	1792.38	1662.82	1733.26	1630.06
AIC	1796.38	1722.81	1759.26	1712.06
BIC	1817.01	1882.23	1828.34	1807.92
Random effect model				
Cluster variance	0.53			
ICC	0.14			
MOR	1.99			
Wasting				
Model fitness				
LR	−446.39	−422.37	−428.64	−409.19
Deviance	892.78	844.74	857.28	818.38
AIC	896.78	904.75	900.37	883.27
BIC	907.41	1064.17	952.35	1118.24
Random effect model				
Cluster variance	0.64			
ICC	0.16			
MOR	2.14			
Underweight				
Model fitness				
LR	−724.58	−673.42	−699.02	−646.66
Deviance	1449.16	1346.84	1398.04	1293.32
AIC	1453.15	1406.84	1424.04	1375.33
BIC	1463.78	1566.25	1493.12	1593.20
Random effect model				
Cluster variance	0.60			
ICC	0.15			
MOR	2.09			
LR test	*X*^2^ = 28.90, *p-*value < 0.001			

LR, Log-likelihood ratio test; AIC, Akaike’s information criterion; BIC, Bayesian information criterion.

Regarding wasting, the null model’s ICC for suffering wasting was 16% of the total variability, with variances occurring between clusters/EA. Furthermore, there was heterogeneity between clusters, as indicated by the null model’s MOR of 2.14. Based on the lowest deviance value (818.38) (Model 3—which includes all explanatory variables), the best-fitted model was selected (Table 3).

The ICC for underweight in the null model was 15% of the total variability, and it was associated with differences between clusters/EA. Furthermore, there was heterogeneity between clusters, as indicated by the null model’s MOR of 2.09. The best-fitted model was chosen based on the lowest deviance value (1,293.32) (Model 3—including all explanatory variables) (Table 3).

### Association between minimum dietary diversity and undernutrition among children aged 6–23 months in Ethiopia

The results of the association between dietary diversity and underweight, wasting, and stunting are shown in Tables 4–6. Using the dietary variety score and minimum dietary diversity as independent variables, this study found that consuming a diverse diet was significantly associated with a lower risk of stunting, wasting, and underweight in children. It was showed that when the number of food groups consumed increased, the risk of experiencing underweight, wasting, and stunting decreased. Therefore, it was evident from both minimum dietary diversity analysis and the dietary diversity score those children who had diverse diets had a lower risk of undernourishment than those who had a less diverse diet. In an adjusted model, children who did receive adequate minimum dietary diversity (MDD) had a significantly lower likelihood of becoming stunted (AOR = 0.68, 95% CI; 0.45, 0.96), wasted (AOR = 0.40, 95% CI = 0.17, 0.97) and underweight (AOR = 0.51, 95% CI; 0.27, 0.95) compared to children who did receive inadequate minimum dietary diversity (MDD).

**Table 4:** Bi-variable and multivariable multilevel logistic regression analysis of the association between minimum dietary diversity and stunting among children aged 6–23 months in Ethiopia, 2024.

Variables	Stunting (HAZ < −2SD)		
Dietary score	*n*	COR (95%CI)	AOR (95%CI)
0	111	Ref.	Ref.
1	167	1.09 (0.79, 1.49)	1.01 (0.67, 1.52)
2	106	1.10 (0.78, 1.57)	0.81 (0.54, 1.23)
3	39	0.68 (0.44, 1.06)	0.66 (0.42, 0.99) *
4	13	0.63 (0.38, 1.06)	0.71 (0.44, 1.16)
5	5	0.91 (0.49, 1.69)	0.47 (0.20, 0.98) *
6	0	–	–
7	2	0.70 (0.22, 2.21)	0.69 (0.27, 1.79)
Minimum Dietary Diversity			
Inadequate (<4 food groups)		Ref.	Ref.
Adequate (≥4 food groups)		0.71 (0.52, 0.97)	0.68 (0.45, 0.96) *
Child characteristics			
Sex of child			
Male		Ref.	Ref.
Female		0.76 (0.60, 0.97)	0.71 (0.55, 0.92) *
Age of child (months)			
6–8		Ref.	Ref.
9–11		1.65 (1.04, 2.61)	1.87 (1.18, 2.99) *
12–17		1.90 (1.28, 2.80)	2.27 (1.52, 3.39) *
18–23		3.19 (2.14, 4.75)	4.08 (2.69, 6.20) *
Maternal characteristics			
Maternal educational level			
No education		Ref.	Ref.
Primary		0.91 (0.70, 1.19)	1.11 (0.81, 1.53)
Secondary		0.55 (0.34, 0.89)	0.72 (0.42, 1.28)
Higher		0.22 (0.11, 0.45)	0.36 (0.17, 0.79) *
Mother’s age			
15–19		Ref.	Ref.
20–24		0.87 (0.52, 1.46)	0.87 (0.52, 1.47)
25–29		0.86 (0.53, 1.42)	0.83 (0.49, 1.39)
30–34		1.25 (0.74, 2.11)	1.26 (0.73, 2.18)
35–39		0.97 (0.54, 1.73)	0.78 (0.42, 1.45)
40–44		0.56 (0.25, 1.23)	0.51 (0.22, 1.19)
ANC visits			
None		Ref.	Ref.
1–3		0.69 (0.49, 0.96)	0.68 (0.47, 0.98) *
4 or more		0.83 (0.61, 1.14)	0.73 (0.54, 1.26)
Place of delivery			
Home		Ref.	Ref.
Health facility		0.75 (0.58, 0.98)	0.69 (0.42, 0.95) *
Other		0.47 (0.11, 0.75)	0.37 (0.15, 0.92) *
Household factors			
Toilet facility			
Improved		Ref.	Ref.
Unimproved		1.26 (0.96, 1.64)	1.21 (0.64, 2.31)
Drinking water source			
Improved		Ref.	Ref.
Unimproved		1.19 (0.91, 1.54)	0.83 (0.44, 1.57)
Wealth index			
Poorest		Ref.	Ref.
Poorer		1.17 (0.81, 1.70)	1.11 (0.73, 1.68)
Middle		1.12 (0.76, 1.66)	1.18 (0.76, 1.84)
Richer		1.16 (0.78, 1.74)	1.25 (0.78, 2.03)
Richest		0.38 (0.26, 0.57)	0.46 (0.25, 0.87) *
Contextual-level factor			
Place of residence			
Urban		Ref.	Ref.
Rural		2.47 (1.74, 3.52)	1.96 (0.59, 2.57)
Geographical regions			
Tigray		Ref.	Ref.
Afar		0.99 (0.55, 1.81)	1.01 (0.53, 1.93)
Amhara		1.46 (0.81, 2.62)	1.51 (0.83, 2.78)
Oromia		0.58 (0.31, 1.05)	0.53 (0.29, 0.99) *
Somali		0.39 (0.19, 0.76)	0.34 (0.16, 0.69) *
Benishangul Gumuz		0.93 (0.50, 1.74)	0.93 (0.49, 1.78)
SNNPR		0.78 (0.44, 1.41)	0.69 (0.37, 1.27)
Gambela		0.44 (0.22, 0.86)	0.44 (0.22, 0.91) *
Harari		0.67 (0.35, 1.29)	0.90 (0.44, 1.83)
Addis Ababa		0.18 (0.07, 0.41)	0.41 (0.16, 1.08)
Dire Dawa		0.50 (0.26, 0.98)	0.74 (0.37, 1.51)

The symbol * indicates that the given variables were statistically significant at 5% level of significance.

**Table 5:** Bi-variable and multivariable multilevel logistic regression analysis of the association between minimum dietary diversity and wasting among children aged 6–23 months in Ethiopia, 2024.

Variables	Wasting (WHZ < -2SD)		
Dietary score	*n*	COR (95%CI)	AOR (95%CI)
0	44	Ref.	Ref.
1	49	0.76 (0.48, 1.20)	0.70 (0.43,1.13)
2	28	0.72 (0.43, 1.23)	0.67 (0.49, 0.96) *
3	8	0.46 (0.20, 1.05)	0.40 (0.15, 0.89) *
4	3	0.39 (0.11, 1.38)	0.22 (0.01, 0.76) *
5	1	0.41 (0.05, 3.35)	0.39 (0.13, 0.74) *
6	0	–	–
7	1	3.12 (0.33, 8.85)	2.04 (0.87, 6.07)
Minimum Dietary Diversity			
Inadequate (<4 food groups)		Ref.	Ref.
Adequate (≥4 food groups)		0.39 (0.22, 0.71)	0.40 (0.17, 0.97) *
Child characteristics			
Sex of child			
Male		Ref.	Ref.
Female		0.70 (0.48, 1.02)	0.69 (0.48, 0.99) *
Age of child (months)			
6–8		Ref.	Ref.
9–11		1.22 (0.63, 2.38)	1.41 (1.11, 2.79) *
12–17		1.21 (0.69, 2.14)	1.40 (0.78, 2.52)
18–23		1.26 (0.70, 2.27)	0.59 (0.06, 0.96) *
Birth order			
1–3		Ref.	Ref.
4–6		1.82 (1.20, 2.74)	1.49 (0.96, 2.32)
>6		1.80 (1.03, 3.14)	1.37 (1.04, 2.21) *
Maternal characteristics			
Maternal educational level			
No education		Ref.	Ref.
Primary		0.55 (0.36, 0.85)	0.48 (0.14, 1.45)
Secondary		0.59 (0.28, 1.22)	0.30 (0.09, 1.51)
Higher		0.28 (0.09, 0.82)	0.26 (0.07, 0.81) *
ANC visits			
None		Ref.	Ref.
1–3		0.67 (0.42, 1.07)	0.55 (0.39, 1.03)
4 or more		0.54 (0.27, 0.90)	0.51 (0.15, 0.89) *
Place of delivery			
Home		Ref.	Ref.
Health facility		0.58 (0.39, 0.88)	0.52 (0.12, 0.93) *
Other		1.09 (0.43, 2.74)	0.49 (0.39, 0.88) *
Household factors			
Wealth index			
Poorest		Ref.	Ref.
Poorer		0.61 (0.35, 1.04)	0.70 (0.39, 1.24)
Middle		0.61 (0.15, 0.92)	0.58 (0.18, 0.99) *
Richer		0.50 (0.27, 0.92)	0.45 (0.33, 1.29)
Richest		0.28 (0.16, 0.91)	0.25 (0.12, 0.84) *

The symbol * indicates that the given variables were statistically significant at 5% level of significance.

**Table 6:** Bi-variable and multivariable multilevel logistic regression analysis of the association between minimum dietary diversity and underweight among children aged 6–23 months in Ethiopia, 2024.

Variables	Underweight (WAZ < -2SD)		
Dietary score	*n*	COR (95%CI)	AOR (95%CI)
0	90	Ref.	Ref.
1	117	0.87 (0.62, 1.23)	0.75 (0.52, 1.08)
2	55	0.58 (0.38, 0.87)	0.53 (0.34, 0.84) *
3	20	0.48 (0.27, 0.86)	0.37 (0.37, 1.61)
4	4	0.21 (0.07, 0.63)	0.11 (0.01, 1.21)
5	2	0.30 (0.06, 1.44)	0.24 (0.02, 1.23)
6	1	0.59 (0.06, 6.03)	0.47 (0.04, 5.07)
7	2	1.60 (0.49, 2.75)	0.80 (0.39, 0.99) *
Minimum Dietary Diversity			
Inadequate (<4 food groups)		Ref.	Ref.
Adequate (≥4 food groups)		0.87 (0.25, 0 0.97)	0.51 (0.27, 0.95) *
Child characteristics			
Sex of child			
Male		Ref.	Ref.
Female		0.59 (0.45, 0.78)	0.54 (0.41, 0.73) *
Age of child (months)			
6–8		Ref.	Ref.
9–11		1.25 (0.75, 2.09)	1.42 (0.84, 2.41)
12–17		1.28 (0.83, 1.99)	1.57 (1.01, 2.46) *
18–23		2.20 (1.42, 3.42)	2.94 (1.85, 4.69) *
Birth order			
1–3		Ref.	Ref.
4–6		1.44 (1.06, 1.96)	1.13 (0.81, 1.58)
>6		1.57 (1.04, 2.38)	1.27 (0.81, 2.01)
Maternal characteristics			
Maternal educational level			
No education		Ref.	Ref.
Primary		0.74 (0.55, 1.01)	1.09 (0.76, 1.57)
Secondary		0.46 (0.26, 0.82)	0.72 (0.37, 1.39)
Higher		0.15 (0.06, 0.41)	0.32 (0.11, 0.92) *
Mother’s age			
15–19		Ref.	Ref.
20–24		0.64 (0.36, 1.15)	0.68 (0.38, 1.23)
25–29		0.77 (0.44, 1.34)	0.76 (0.42, 1.36)
30–34		1.24 (0.70, 2.19)	1.07 (0.55, 2.06)
35–39		1.02 (0.54, 1.93)	0.75 (0.35, 1.61)
40–44		1.00 (0.44, 2.29)	0.77 (0.29, 2.05)
ANC visits			
None		Ref.	Ref.
1–3		0.70 (0.49, 1.02)	0.78 (0.53, 1.17)
4 or more		0.77 (0.39, 0.91)	0.68 (0.44, 0.94) *
Place of delivery			
Home		Ref.	Ref.
Health facility		0.54 (0.40, 0.73)	0.48 (0.24, 0.61) *
Other		0.55 (0.25, 1.25)	0.50 (0.32, 1.13)
Household factors			
Toilet facility			
Improved		Ref.	Ref.
Unimproved		1.39 (1.03, 1.89)	1.20 (0.57, 2.53)
Drinking water source			
Improved		Ref.	Ref.
Unimproved		1.38 (1.02, 1.87)	0.91 (0.43, 1.96)
Wealth index			
Poorest		Ref.	Ref.
Poorer		0.89 (0.59, 1.35)	0.94 (0.59, 1.47)
Middle		0.57 (0.36, 0.90)	0.66 (0.40, 1.11)
Richer		0.63 (0.39, 1.21)	0.57 (0.24, 1.02)
Richest		0.41 (0.19, 0.49)	0.38 (0.19, 0 0.77) *
Contextual-level factor			
Place of residence			
Urban		Ref.	Ref.
Rural		1.61 (1.08, 2.39)	1.18 (1.02, 2.15) *
Geographical regions			
Tigray		Ref.	Ref.
Afar		0.55 (0.29, 1.05)	0.30 (0.15, 0.60) *
Amhara		0.82 (0.44, 1.52)	0.79 (0.43, 1.48)
Oromia		0.22 (0.11, 0.44)	0.17 (0.08, 0.36) *
Somali		0.40 (0.20, 0.80)	0.17 (0.08, 0.36) *
Benishangul Gumuz		0.65 (0.34, 1.25)	0.58 (0.29, 1.12)
SNNPR		0.41 (0.21, 0.77)	0.29 (0.15, 0.56) *
Gambela		0.61 (0.31, 1.22)	0.46 (0.22, 0.93) *
Harari		0.31 (0.14, 0.65)	0.29 (0.13, 0.64) *
Addis Ababa		0.04 (0.01, 0.19)	0.06 (0.01, 0.26) *
Dire Dawa		0.38 (0.18, 0.78)	0.38 (0.18, 0.81) *

The symbol * indicates that the given variables were statistically significant at 5% level of significance.

On multivariable analysis (AOR); minimum dietary diversity, sex of child, age of child, maternal education, ANC visit, place of delivery, wealth index, and regions were significantly associated with stunting. Furthermore, minimum dietary diversity, sex of child, age of child, birth order, maternal education ANC visit, place of delivery, and wealth index were significantly associated with wasting. In addition, minimum dietary diversity, sex of child, age of child, maternal education, ANC visit, wealth index, residence, and regions were significantly associated with underweight (Tables 4–6).

Children with adequate MDD had a 32% lower chance of being stunted (AOR = 0.68, 95% CI = 0.45, 0.96) than those with inadequate MDD, according to the results of the full model (model 3).

Additionally, it was found that, in comparison to their male counterparts, female children had a 29% lower risk of being stunted (AOR = 0.71, 95% CI = 0.55, 0.92). Additionally, mothers who attended 1–3 ANC had a 32% reduced likelihood (AOR = 0.68, 95% CI = 0.47, 0.98) of having a stunted child, compared to those who did not attend ANC visits. Moreover, children from households with the richest wealth index had a 54% lower chance of stunting (AOR = 0.46, 95% CI = 0.25, 0.87) than children from households with the poorest wealth index (Table 4).

Based on the findings of the association between minimum dietary diversity and wasting, the complete model (model 3) shows again that, having an adequate MDD presented 60% reduced odds of wasting among children (AOR = 0.40, 95% CI = 0.17, 0.97), compared to those who had inadequate MDD. Additionally, birth order 6 and above increased the risk of wasting (AOR = 1.37, 95% CI = 1.04, 2.21) in children. Moreover, children aged 9–11 months were more likely to be wasted by 41% (AOR = 1.41, 95% CI = 1.11, 2.79) compared to those whose age group was 6–8 months. Children of mothers with higher education had a significantly reduced risk of wasting.

In addition, children from mothers with higher education had a 74% reduced risk of wasting (AOR = 0.26, 95% CI = 0.07, 0.81), as compared to children from mothers with uneducated. Children from the households with the richest wealth index also showed less likely to be wasted (AOR = 0.25, 95% CI = 0.12, 0.84) when compared to children from the households with the poorest wealth index. Moreover, attending at least four antenatal care visits (ANC) was associated with a 49% lower risk of wasting in children (AOR = 0.51, 95% CI = 0.15, 0.89) (Table 5).

The results of model 3 with all explanatory variables on the association between minimum dietary diversity and underweight showed that children with adequate MDD had a significant reduction in underweight risk by 49% (AOR = 0.51, 95% CI = 0.27, 0.95) compared to those with inadequate MDD. Additionally, children aged 18–23 months were nearly 3 times more likely to be underweight (AOR = 2.94, 95% CI = 1.85, 4.69) compared to those whose age group was 6–8 months. Moreover, Children delivered at a health facility had a significantly lower risk of underweight by 52% (AOR = 0.48, 95% CI = 0.24, 0.61), as compared to children who delivered at home. Children living in rural areas were more likely to be underweight (AOR = 1.18, 95% CI = 1.02, 2.15) compared to their urban counterparts (Table 6).

## Discussion

This study examined the association between minimum dietary diversity and undernutrition (stunting, wasting, and underweight) among children aged 6–23 months in Ethiopia. We also examined the association between other child and maternal characteristics, household, and contextual factors, and undernutrition. The study found a significant association between minimum dietary diversity and stunting; in particular, having adequate dietary diversity reduced the likelihood of stunting among children. Moreover, the study also revealed a significant association between dietary diversity and being underweight; that is, an adequately diversified diet reduced the likelihood of being underweight. Finally, the study revealed a substantial association between dietary diversity and wasting, emphasizing that adequate dietary diversity significantly reduced the odds of wasting among children in Ethiopia. These results are similar to findings by other studies in SSA (4, 5, 42) and findings reported from other developing countries like Ghana (14), Tanzania (16), Burkina Faso (41), Ethiopia (9, 19, 21, 22) and Kenya (46).

Our results suggest that increased dietary diversity of complementary foods in Ethiopia could contribute to a decrease in undernutrition. This is supported by the fact that dietary diversity is a good predictor of dietary quality and micronutrient density in children (16, 47, 48). In light of this, policymakers should consider dietary diversity as one of the key elements in their efforts to improve the nation’s children’s nutritional status. Additionally, the study (49) reported that the nutritional status of children is influenced by their diet and an increase in consumption of diversified foods could reduce undernutrition including underweight among children.

The findings on the association between MDD and stunting are similar to findings by other studies from Ethiopia (9, 19) and Indonesia (3). Different factors could explain this finding. A major factor for explaining this finding is that, when children have an adequate dietary diversity, it means they receive foods from a minimum of four of the seven groups of foods-namely grains, roots, and tubers; legumes and nuts; dairy products; flesh foods (meat, fish, poultry, and organ meats); eggs; vitamin-A rich fruits and vegetables; other fruits and vegetables (22, 50). Receiving foods from these groups provides children with sufficient nutrients needed to reduce the risk of stunting (3) as found in this study.

Likewise, an association between MDD and underweight was found, which is in line with results from another study conducted in SSA (42). A major factor that explains this finding is similar to the explanation above, where adequate dietary diversity means receiving foods from a minimum of four of the seven food groups (50), which has been posited by Walingo and Ekesa (51) to reduce the risk of underweight among children. Additionally, Khamis et al. (16) reported that the nutritional status of children is influenced by their diet and an increase in consumption of diversified foods could reduce undernutrition including underweight among children.

Results from research conducted in South Ethiopia (52), South Africa (53), Burundi, and the Democratic Republic of Congo (54) are consistent with the association found in this study. Importantly, adequate dietary diversity is receiving foods from a minimum of four of the seven groups of foods (19, 50) and this will provide children with the sufficient nutrients needed to reduce the likelihood of wasting among them (14). However, findings from another study conducted in Burkina Faso (41) were inconsistent with those of this study on the association between wasting and adequate MDD. According to the study conducted in Burkina Faso (41), dietary diversity was not associated with wasting among children. The difference in the findings could be due to the different methods used and the previous study was based on a randomized control trial and included children aged 6–59 months, whereas the present study is a secondary data analysis of cross-sectional MDHS data from Ethiopia.

According to other findings, a child’s age increased the likelihood that they would experience undernutrition (stunting, wasting, and underweight). Children in the aged 18–23 months were more likely to experience malnutrition than children in the aged 6–8-months. The findings of this study on the association between the age of a child and undernutrition are supported by another study (42). The current study also found that; being female, children with mothers with higher education, attending 4 or more ANC visits, delivered at health facilities, and children from households with the richest wealth index decreased the odds of being undernourished. These findings are consistent with the studies conducted in SSA (42, 55).

Therefore, our study highlights the critical role of minimum dietary diversity in addressing undernutrition among children aged 6–23 months in Ethiopia, as revealed through multilevel mixed-effect logistic regression analysis based on the 2019 Ethiopian Mini Demographic and Health Survey. Future research endeavors should delve deeper into understanding the socio-economic and cultural determinants of dietary diversity and undernutrition in Ethiopia. Moreover, targeted interventions focusing on promoting diverse and nutritious diets for children, along with improving access to essential healthcare services, are imperative to mitigate the burden of undernutrition and ensure the well-being of Ethiopia’s youngest population.

### Strengths and limitations of this study

The study has several limitations. First, the analysis was limited to the variables available in the 2019 Mini Demographic and Health Survey dataset, as the study relied on secondary data. Consequently, the conclusions and interpretations should be understood within the context of the included variables. The study’s cross-sectional design, based on the 2019 Mini Demographic and Health Survey (MDHS), captures data at a single point in time, which restricts the ability to establish causal relationships between minimum dietary diversity and undernutrition. The observed associations may not necessarily imply cause and effect. Additionally, the reliance on caregivers’ self-reported data for dietary diversity could introduce recall bias or social desirability bias, potentially affecting the accuracy of the reported dietary practices. Lastly, since the 2019 Mini Demographic and Health Survey did not include data on calorie consumption, our study could not account for total caloric intake, which may influence the study’s conclusions. The study has merits despite its limitations. One of the study’s main strengths is the utilization of high sample sizes and nationally representative data. Additionally, the study’s results fill a gap in the research on dietary diversification and undernutrition in Ethiopia.

## Conclusion

According to the findings, nearly one-tenth of study participants had an adequate minimum dietary diversity, approximately one-fourth of study participants had stunted, about 5% of children were wasted and nearly one-seventh of children were underweight. The study also found that there is a significant association between minimum dietary diversity and undernutrition (stunting, wasting, and underweight) among children aged 6–23 months in Ethiopia. Significant associations between these undernutrition measures and other important variables were also found. To improve infant and young child feeding (IYCF) behaviors, particularly complementary feeding practices among Ethiopian children aged 6–23 months, more nutrition-specific interventions are therefore required, as well as the reinforcement of current programs.

In addition to addressing the obstacles to appropriate feeding practices, these interventions should incorporate evidence-based educational models on proper feeding practices for childbearing women. Regions with low prevalence of minimum dietary diversity and high rates of undernutrition should be the primary target of these efforts. It is recommended that future research investigate the impact of distinct food groups found in a diverse diet on children’s nutritional status.

## Data Availability

The original contributions presented in the study are included in the article/supplementary material, further inquiries can be directed to the corresponding author.
